# Dietary fiber intake and pancreatic cancer risk: a meta-analysis of epidemiologic studies

**DOI:** 10.1038/srep10834

**Published:** 2015-06-02

**Authors:** Chun-Hui Wang, Chong Qiao, Ruo-Chen Wang, Wen-Ping Zhou

**Affiliations:** 1 Department of Hepatobiliary Surgery, General Hospital of Shenyang Military Region, Shenyang, China; 2 Department of Obstetrics and Gynecology, Shengjing Hospital of China Medical University, Shenyang, China; 3 Liaoning Province Shiyan High School, Shenyang, China

## Abstract

Evidence on the association between dietary fiber intake and pancreatic cancer risk has been controversial. Therefore, we carried out this meta-analysis to summarize available evidence from epidemiologic studies on this point. Relevant studies were identified by searching PubMed, Embase and Web of Science databases as well as by reviewing the rence lists of relevant articles. Random or fixed-effects model was used to calculate the summary risk estimates and 95% confidence intervals (CIs). This meta-analysis included one cohort and thirteen case-control studies which involving a total of 3287 subjects with pancreatic cancer. After summarizing the risk estimates of these studies, we yielded a significant association between dietary fiber intake and pancreatic cancer risk among case-control studies (odds ratio = 0.54; 95%CI = 0.44–0.67; *I*^2^ = 41.4%; *P* = 0.043) but a non-significant result in cohort study (hazard ratio = 1.01; 95%CI = 0.59–1.74). Additionally, significant inverse associations were observed when we carried out the stratify analyses by the study characteristics and adjustment for potential confounders among case-control studies. Given only one cohort study included in the present meta-analysis, further prospective-designed studies should validate our findings and report more detail results, including those for subtypes of fiber, the risk estimates which corrected the impact of measurement errors and fully adjust for the potential confounders.

As one of the most fatal types of cancer, about 0.3 million new cases of pancreatic cancer were diagnosed and nearly the same number of patients dead from this disease in 2012 worldwide^1^. Prognosis of this disease is extremely poor, with a 1-year survival rate of 25% and a 5-year survival rate below 5%^2^. Given no effective screening at present, priority should be given to identification of more modifiable risk factors and prevention of the disease.

Since dietary factors may partly play a potential role in the etiology of pancreatic cancer^3^,^4^, understanding this role would bring substantial clinical and public health benefits. Nevertheless, no convincing dietary risk factors for pancreatic cancer have been established by the report from the World Cancer Research Fund and the American Institute for Cancer Research (WCRF/AICR) reported in 2007^4^.The possibility of an association between dietary fiber intake and pancreatic cancer risk has received considerable interest and has been investigated intensively during the recent two decades. Although several plausible biologic mechanisms have been hypothesized to underlie the possible protective effect of fiber intake, the evidence from epidemiologic studies has been inconclusive. Additionally, besides total fiber, several studies suggested that the beneficial effects might be only limited in soluble and insoluble fiber^5^,^6^. To our knowledge, a systematical and comprehensive assessment of the association between dietary fiber intake and pancreatic cancer risk has not been reported before. Thus, to clarify the aforementioned issues, we carried out the present meta-analysis based on published epidemiologic studies.

## Results

### Search results, study characteristics, and quality assessment

**Figure 1:**
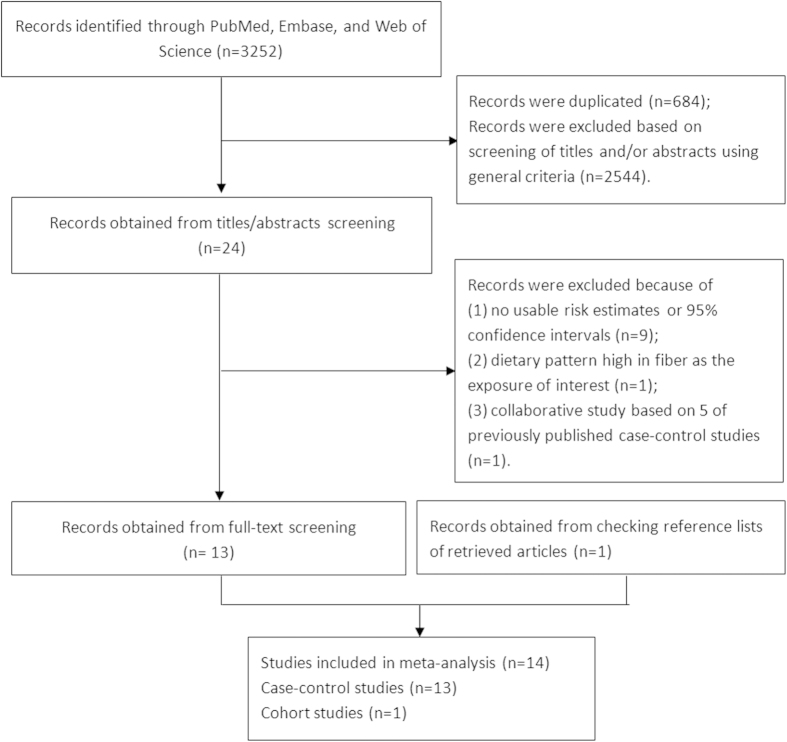
Flow-chart of study selection.

The search identified a total of 1,953 articles for which the titles and abstracts were scanned to determine potential eligibility for inclusion. After a selection process (Fig. 1), 14 studies^3^,^5^,^6^,^7^,^8^,^9^,^10^,^11^,^12^,^13^,^14^,^15^,^16^,^17^ fulfilled our inclusion criteria and were included in this meta-analysis (1 cohort study and 13 case-control studies). Although Ji *et al.*^10^ and Lyon *et al.*^12^ have provided the risk estimates separately by gender, they were treated as two studies in all. These 14 studies cumulatively reported 3,287 pancreatic cancer cases.

Table 1 summarizes the characteristics of these included studies which were conducted in the North America (n = 6), Europe (n = 5), and others (including Asia and Australia) (n = 3). All studies conducted multivariable analyses, adjusted for age and cigarette smoking, and most studies also adjusted for gender (n = 13) and energy intake (n = 13). Fewer studies controlled for body mass index (n = 3), alcohol drinking (n = 5), and history of diabetes (n = 3). The dietary habits of majority of included studies were investigated to the period 1-5 years before cancer diagnosis for cases or interview for controls, except for the study reported by Lyon *et al.*^12^. Seven case-control studies^10^,^12^,^13^,^14^,^15^,^16^,^17^ used proxy respondents of cases or controls when collecting the information of diet habits.

The information of study quality are summarized in Supplementary Table S1 and Supplementary Table S2. Briefly, three case-control studies^5^,^6^,^11^ were not assigned a star in the column of “selection of control subjects” because these studies utilized hospital-based controls. One cohort study and five case-control studies^5^,^6^,^8^,^9^,^11^,^13^ were assigned two stars in the column of “control for important factor or additional factor” because they adjusted for more than two important confounders in the primary analyses. Six case-control studies^10^,^11^,^12^,^15^,^16^,^17^ were not assigned a star in the column of “exposure assessment” because their questionnaires were not validated. Nine case-control studies^3^,^6^,^10^,^12^,^13^,^14^,^15^,^16^,^17^ were not assigned a star in the column of “non-response rate” because the response rate between cases and controls were significantly differenced.

### Dietary fiber and pancreatic cancer risk

**Figure 2:**
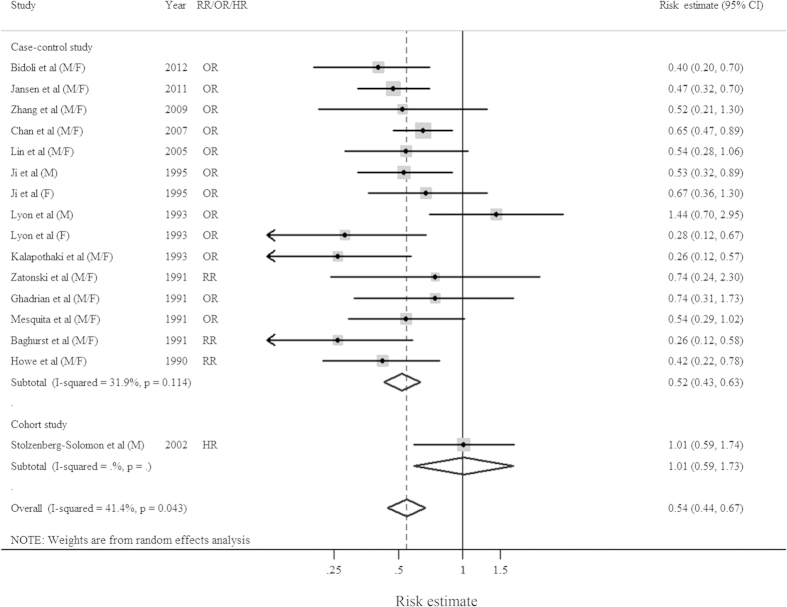
Forest plots (random effect model) of meta-analysis on the relationship between fiber intake and pancreatic cancer risk by study design. Squares indicate study-specific risk estimates (size of the square reflects the study-specific statistical weight); horizontal lines indicate 95% CIs; diamond indicates the summary risk estimate with its 95% CI. F: female; HR: hazard ratio; M: male; OR: odds ratio; RR: relative risk.

The multivariable-adjusted risk estimates for each study and the summary risk estimates for the highest *versus* the lowest categories of dietary fiber intake by study design are shown in Fig. 2. Diet high in fiber was associated with a statistically significant reduction in pancreatic cancer risk among case-control studies, with the corresponding summary risk estimate of 0.52 (95%CI = 0.43–0.63; *I*^2^ = 31.9%, *P* = 0.114). However, there is no association between fiber intake and pancreatic cancer risk in cohort study, though only one study was included (Table 2 and Fig. 2). There was no evidence of publication bias, both quantitatively (*P* = 0.495 for Egger’s test and *P* = 0.787 for Begg’s test) and qualitatively, on visual inspection of the funnel plot.

### Subgroup and sensitivity analysis

Since cohort study and case-control study were two different study designs and only one cohort study was found in the literature search, we excluded this cohort study in the subgroup analyses. When we carried out the stratified analysis by fiber type, significant results were observed in soluble fiber and insoluble fiber but the result showed borderline significance in crude fiber. Furthermore, table 2 shows the associations between fiber intake and pancreatic cancer risk in pre-planned subgroup meta-analyses stratified by type of control subjects, proxy respondent, exposure assessment method, geographic location, and adjustment for potential confounders. Significant inverse associations of fiber intake on pancreatic cancer were observed among almost all the strata of between-study subgroup analyses.

In a sensitivity analysis in which we removed one study at a time and analyzed the rest, the summary risk estimates ranged from 0.53 (95%CI = 0.46–0.62, *I*^2^ = 31.9%) after excluding the study by Stolzenberg-Solomon *et al.*^9^ to 0.57 (95%CI = 0.46–0.69, *I*^2^ = 36.0%) after excluding the study by Baghurst *et al.*^13^. Additionally, we excluded the study by Kalapthaki *et al.*^11^ in which risk estimate and 95%CI was recalculated. The summary risk estimate from this sensitivity analysis was 0.57 (95%CI = 0.46–0.69, *I*^2^ = 35.8%) which was similar to the main finding.

## Discussion

To the best of our knowledge, this is the first meta-analysis to summarize the evidence between total and different types of fiber intake and risk of pancreatic cancer. In our meta-analysis, increased fiber intake is associated with a reduced risk of pancreatic cancer in case-control studies but not in cohort study. The findings partly support the hypothesis that diet high in fiber may provide protection against pancreatic cancer.

The report of WCRF/AICR in 2007 concluded that there was “limited-no conclusion” evidence between foods containing dietary fiber and pancreatic cancer risk which was based on five case-control studies and one cohort study^4^. After that, several additional case-control studies^5^,^6^,^7^,^8^ were published in the recent years. Studies by Bidoli *et al.*^5^, Jansen *et al.*^6^, Zhang *et al.*^7^, and Chan *et al.*^8^, including a total of 1428 pancreatic cancer cases, accounted for over 43% of the patients in the studies included in this meta-analysis. Compared with the report of WCRF/AICR, this meta-analysis includes more pancreatic cancer cases and updates the evidence between dietary fiber intake and risk of pancreatic cancer.

Quality scoring might not only submerge important information by combining disparate study features into a single score but introduce somewhat arbitrary subjective element into the analysis^18^,^19^,^20^. Therefore, although the Newcastle-Ottawa Scale (NOS) was used to assess the quality of included studies, we did not score these included studies or categorize them into high or low quality according to the scores. The results of quality assessment demonstrated that compared with prospective study, case-control studies including in this meta-analysis were less likely to use validated food frequency questionnaire and adjust for potential confounders but more likely to have significantly difference in non-response rate between cases and controls (Supplementary Table 1 and 2). Besides, because information on exposures is collected before the diagnosis of the disease, cohort studies are less susceptible to recall bias than case-control studies. Given this, several biases (e.g. selection bias, information bias, or confounding bias) might be introduced in their primary analyses. Additionally, since the majority of included studies were case-control studies (13/14), we carried out stratified analyses to explore the sources of heterogeneity by excluding the only one cohort study. Therefore, the findings of the stratified analyses on the basis of case-control studies should be interpreted with cautious. Population-based control subjects were more likely to provide a relatively exact estimate of the exposure than hospital-based controls^3^. We found a slightly attenuated point estimate among the population-based case-control studies compared with the hospital-based case-control studies (Table 2). Furthermore, when stratified by the geographic location, we found that the point estimate for Europeans was slightly attenuated than North Americans and Asians, which might be partly attributed to the different amount of fiber intake. However, restricted by the limited included studies in the stratified analysis, this issue need further investigation.

Although the exact biologic mechanisms underlying the aforementioned inverse association are not fully understood, several biologic plausible reasons might have been proposed to partly explain the protective role of dietary fiber. Foods rich in fiber are known to have several anti-carcinogenic properties, such as the ability to lower levels of circulating markers of inflammation which may be involved in pancreatic cancer initiation and progression^21^,^22^, and the ability to improve insulin metabolism by modulating hormonal pathways linked to pancreatic carcinogenesis which have been associated with cancer promotion^23^,^24^. Several experimental studies suggested that inositol hexaphosphate, a naturally occurring molecule found in high-fiber foods, was compound that has been shown to demonstrate anti-proliferative effects, resulting in the inhibition of pancreatic cancer cell growth^25^,^26^. Since these biologic mechanisms are speculative and there are limited experimental data available, further *in vivo* and *in vitro* studies are warranted to shed light on the underlying mechanisms between total and different types of fiber intake and pancreatic cancer risk.

Our study has several strengths. This is the first meta-analysis focused on the relationship between dietary fiber intake and risk of pancreatic cancer. All studies ascertain outcomes using histological findings. Compared to these included studies, the present meta-analysis includes a total of 3,287 cases, which significantly increase the statistical power of the main analysis. Additionally, the summary risk estimate remains stable and robust in the subgroup and sensitivity analyses.

Despite the clear strengths of this meta-analysis, limitations of the present meta-analysis also require considerations. Diets high in fiber may be related to other behaviors including smoking, alcohol drinking, overweight and obesity, diabetes mellitus, and intake of total energy, which could possibly confound the observed associations. However, the summary risk estimates did not change materially in subgroup analyses whether adjusted for major potential confounders, such as body mass index^5^,^6^,^8^, diabetes mellitus^5^,^8^,^11^,alcohol drinking^5^,^6^,^7^,^12^,^13^ and total energy intake^3^,^5^,^6^,^7^,^8^,^9^,^10^,^11^,^13^,^14^,^15^,^16^,^17^. Since limited studies were included in some of the analyses (Table 2), prudence still should be used when interpreting these findings. Second, accurate assessment of dietary fiber intake and other food constituents is a challenge which may bias effect estimates^27^. A previous meta-analysis focused on fiber intake and colorectal cancer suggested that the different definition of dietary fiber between included studies might be concerned when exploring the source of the heterogeneity. However, only one study^5^ mentioned the Englyst definition of fiber in their methods. Compared to the Association of Official Analytical Chemists method which includes some starch as dietary fibre, the Englyst definition distinguishes non-starch polysaccharides from starch^27^. In addition, although slightly stronger risk estimates without heterogeneity was observed among these studies using validated food frequency questionnaire when we carried out the stratified analysis by exposure assessment (Table 2), none of the studies included in the present meta-analysis made any corrections for measurement errors. Measurement errors would, however, most likely result in bias toward the null which suggests that our result for fiber intake and pancreatic cancer risk is likely to be underestimation of the true underlying risk. Further studies should consider correction for measurement error in the analyses. Third, only Stolzenberg-Solomon *et al.*^9^ reported the results of fiber intake and pancreatic cancer risk based on prospective design. Although the results of older male heavy smokers may not be generalizable to nonsmoking populations, compared to case-control study, prospective cohort study are less susceptible to some biases (e.g., selection bias and recall bias)^28^. Preclinical symptoms of pancreatic cancer (e.g., anorexia and indigestion) may influence the dietary habits such as fiber intake, which may lead to reverse causality in epidemiological studies. However, since the short latency of pancreatic cancer and diet pattern was evaluated for 1-5 years before cancer diagnosis among the majority of included studies which have already concerned about the possibility of diet change caused by the preclinical symptoms^5^. Therefore, reverse causality may not be a major problem in this study. Besides, given the poor survival of pancreatic cancer, the questionnaires or interviews of some early studies^12^,^13^,^14^,^15^,^16^,^17^ were completed by proxy respondents which may also bias the risk estimates. For example, Lyon *et al.*^12^ found that the response rate for cases was greater than that for controls which might result from the different willingness of the next-of-kin of the cases to participate in a study compared with proxy respondents of randomly selected controls. Additionally, most proxy respondents of their study were spouses and recall of spouses diet may differ by sex of the surrogate respondent. This might lead to the different risk estimates of dietary intake between men and women including fiber. Although the result of meta-regression showed no difference between studies whether using proxy respondents (Table 2), notably, we found a significant heterogeneity among studies using proxy respondent which might contribute heterogeneity to the main result. Last, we found the results of trend analysis of many included studies were statistical significant^5^,^6^,^8^,^10^,^11^,^13^,^17^, but due to only four included studies provided enough information for a dose-response meta-analysis^5^,^6^,^7^,^8^, future studies and pooled analysis are warranted to investigate whether there is a non-linear relationship between fiber intake and pancreatic cancer risk.

In conclusion, this meta-analysis suggests that a high intake of dietary fiber is associated with a reduced risk of pancreatic cancer in case-control studies but not in cohort study. Further studies, especially prospective-designed studies should validate our finding and report more detail results, including those for subtypes of fiber, the risk estimates which corrected the impact of measurement errors, and fully adjust for the potential confounders in the future.

## Materials and Methods

### Search Strategy

In this meta-analysis, we followed the guidelines developed by the Meta-analysis Of Observational Studies in Epidemiology group (MOOSE)^29^. Two authors (C-HW and CQ) performed a systematic literature search in the MEDLINE (PubMed; http://www.ncbi.nlm.nih.gov/pubmed), Embase and Web of Science databases through August, 2014 without limitations by using the following search key words: (diet or dietary or fiber or fibre) and (pancreatic or pancreas) and (cancer or neoplasm). Furthermore, bibliographies of relevant studies were hand-searched for additional publications.

### Study Selection

The title and abstract of studies identified in the search were reviewed by two authors (C-HW and CQ) to exclude studies that did not answer the research question of interest. Studies considered in this meta-analysis should meet the following inclusion criteria: the study (1) had a observational study design (e.g., cohort, case-cohort, nested case-control, or case-control study); (2) clearly defined dietary fiber as the exposure of interest; (3) reported pancreatic cancer as the outcome of interest; and (4) reported relative risks (RRs), odds ratios (ORs), and hazard ratios (HRs) with 95% confidence intervals (CIs) or provided data for their calculation. Inclusion was not otherwise restricted by study size, language, or publication type. If multiple articles were on the same study population, the one with more informative data was selected.

### Data Extraction

Data were independently abstracted onto a standardized form by two authors (C-HW and CQ). Conflicts in data abstraction were resolved by consensus, referring back to the original article. The following data were collected from each study: first author’s last name, year of publication, country of the study population, study design, sex, study period, number of cases and controls or cohort size, fiber intake categories and methods of dietary assessment, risk estimates with their 95% CIs for the highest *versus* lowest category, and factors matched by or adjusted for in the design or data analysis. From each study, we extracted the risk estimates that reflected the greatest degree of control for potential confounders. Only two studies^10^,^12^ provided information stratified by sex and so each subgroup is included separately. For study reported by Kalapothaki *et al.*^11^ that only reported the results by 1 standard deviation increment of dietary fiber intake, we converted the reported risk estimates into a standard scale of effect to compare persons with dietary fiber intakes in the top quintile with persons whose intakes were in the bottom quintile^30^,^31^, which was the most common in the included studies^5^,^8^,^9^,^14^,^17^. Subsequently, the inverse-variance method was used to summary the converted risk estimates of the different control populations of this study^32^.

### Quality assessment

The NOS includes 3 quality parameters for case-control or cohort studies: the selection of study groups, comparability of groups and ascertainment of either the exposure or outcome of interest was used by two independent researchers (C-HW and CQ) to assess study quality^32^,^33^,^34^.

### Statistical analysis

As the absolute risk of pancreatic cancer is low, as well as only three included case-control studies^13^,^16^,^17^ which reported the risk estimate as RR, therefore, we reported the risk estimates from case-control studies as the OR for simplicity. For studies^10^,^12^ that reported the results separately by gender, we combined these results with the rest of the studies instead of using a fixed-effect model to obtain an overall combined estimate aforehand^32^,^35^. We used the fixed or random-effects model^36^,^37^ to calculate summary risk estimates and 95% CIs for the highest *versus* lowest categories of dietary fiber according to the result of heterogeneity test. In assessing heterogeneity among studies, we used the *I*^2^ statistics which values represent the amount of total variation explained by variation among studies, with a value of greater than 50% considered to indicate severe heterogeneity and a value of less than 25% indicating the absence of significant heterogeneity^37^. Small study bias, such as publication bias (publication bias considered present if *P* < 0.1), was evaluated via Egger’s linear regression^38^, Begg’s rank-correlation methods^39^, and funnel plots. In order to investigate possible sources of heterogeneity among studies, we not only carried out the stratified analyses by study design and fiber type, but also conducted subgroups analyses according to potentially relevant factors: type of control subjects (population-based *versus* hospital-based), proxy respondent (yes *versus* no), exposure assessment (validated questionnaire *versus* others), geographic location (North America, Europe, Asia, and Oceania), and confounders that were adjusted for the following: body mass index, sex, diabetes mellitus, total energy intake, and alcohol drinking. Heterogeneity between subgroups was evaluated by meta-regression. At last, we conducted sensitivity analyses by deleting each study in turn to reflect the influence of individual data sets on the overall estimate. For all tests, a probability level <0.05 was considered statistically significant. All statistical analyses were conducted by using Stata software (version 11.2; StataCorp).

## Additional Information

**How to cite this article**: Wang, C.-H. *et al*. Dietary fiber intake and pancreatic cancer risk: a meta-analysis of epidemiologic studies. *Sci. Rep.* **5**, 10834; doi: 10.1038/srep10834 (2015).

## Supplementary Material

Supplementary Information

## Figures and Tables

**Table 1:** Characteristics of studies of fiber intake and pancreatic cancer risk.

**First Author, (Reference), Year, Country, Study design**	**Sex**	**Study Period**	**Case/Control (Cohort size)**	**Fiber categories (dietary assessment)**	**Risk Estimates (95% CI)**	**Matched/Adjusted factors**
Bidoli *et al.*^5^, 2011, Italy, HB	M/F	1991-2008	326/652	Total: Q5 *versus* Q1	0.4 (0.2–0.7)	Age, sex, center, period of interview, BMI, education, cigarette smoking, alcohol consumption, self-reported history of diabetes, dietary folate and total energy intake
				Soluble: Q5 *versus* Q1	0.4 (0.2–0.7)	
				Insoluble: Q5 *versus* Q1	0.5 (0.3–0.8)	
				(Validated FFQ)	Odds ratio	
Jansen *et al.*^6^, 2011, USA, HB	M/F	2004-2009	384/983	Total: Q5 *versus* Q1	0.47 (0.32–0.70)	Age, sex, region of residence, energy, cigarette smoking, BMI, and drinks of alcohol
				Soluble: Q5 *versus* Q1	0.58 (0.39–0.86)	
				Insoluble: Q5 *versus* Q1	0.48 (0.33–0.71)	
				(Validated DHQ)	Odds ratio	
Zhang *et al.*^7^, 2009, USA, PB	M/F	1994-1998	186/554	Total: Q4 *versus* Q1	0.52 (0.21–1.30)	Age, sex, race, education, cigarette smoking, alcohol, physical activity and intakes of all other dietary factors
				(Validated FFQ)	Odds ratio	
Chan *et al.*^8^, 2007, USA, PB	M/F	1995-1999	532/1701	Total: Q4 *versus* Q1	0.65 (0.47–0.89)	Age, sex, BMI, race, education, cigarette smoking, history of diabetes, and energy intake
				Crude: Q4 *versus* Q1	0.70 (0.51–0.97)	
				(Validated FFQ)	Odds ratio	
Lin *et al.*^3^, 2005, Japan, PB	M/F	2000-2002	109/218	Total: T3 *versus* T1	0.54 (0.28–1.06)	Age, cigarette smoking, and energy intake
				(Validated FFQ)	Odds ratio	
Stolzenberg-Solomon *et al.*^9^, 2002, Finland, Cohort	M	1985-1997	163/27,111	Total: Q5 *versus* Q1	1.01 (0.59–1.74)	Age, years of cigarette smoking and energy intake
				Soluble: Q5 *versus* Q1	1.02 (0.56–1.63)	
				Insoluble: Q5 *versus* Q1	0.95 (0.57–1.60)	
				(Validated DHQ)	Hazard ratio	
Ji *et al.*^10^, 1995, China, PB	M/F	1970-1990	451/1552	Total: Q4 *versus* Q1 (M)	0.53 (0.32–0.89)	Age, income, cigarette smoking, green tea drinking (females only), response status, and total calories intake
				Total: Q4 *versus* Q1 (F)	0.67 (0.36–1.30)	
				(FFQ)	Odds ratio	
Lyon *et al.*^12^, 1993, USA, PB	M/F	1984-1987	149/363	Total: High *versus* Low (M)	1.44 (0.70–2.95)	Age, cigarette smoking, and intake of coffee and alcohol
				Total: High *versus* Low (F)	0.28 (0.12–0.67)	
				(FFQ)	Odds ratio	
Kalapothaki *et al.*^11^,* 1993, Greece, HB	M/F	1991-1992	181/181	Crude: Q5 *versus* Q1	0.26 (0.12–0.57)	Age, sex, hospital, past residence, years of schooling, cigarette smoking, diabetes mellitus, total energy, carbohydrate, protein, and fat intake
				(DHQ)	Odds ratio	
Zatonski *et al.*^16^, 1991, Poland, PB	M/F	1985-1988	110/195	Total: Q4 *versus* Q1	0.74 (0.24–2.30)	Age, sex, residence, cigarette smoking and energy intake
				(DHQ)	Relative Risk	
Ghadrian *et al.*^15^, 1991, Canada, PB	M/F	1984-1988	179/239	Total: Q4 *versus* Q1	0.74 (0.31–1.73)	Age, sex, cigarette smoking status, response and energy intake
				Crude: Q4 *versus* Q1	0.81 (0.35–1.85)	
				(FFQ)	Odds ratio	
Mesquita *et al.*^14^, 1991, Netherlands, PB	M/F	1984-1988	164/480	Total: Q5 *versus* Q1	0.54 (0.29–1.02)	Age, sex, response status, cigarette smoking status and energy intake
				(Validated FFQ)	Odds ratio	
Baghurst *et al.*^13^, 1991, Australia, PB	M/F	1984-1987	104/253	Total: Q4 *versus* Q1	0.26 (0.12–0.58)	Age, sex, total energy intake, alcohol and cigarette usage
				(Validated	Relative	
				FFQ)	Risk	
Howe *et al.*^17^, 1990, Canada, PB	M/F	1983-1986	249/505	Total: Q5 *versus* Q1	0.42 (0.22–0.78)	Age, sex, caloric intake, and cigarette smoking
				(Validated DHQ)	Relative Risk	

BMI, body mass index; DHQ, dietary history questionnaire; F, female; FFQ, food frequency questionnaire; HB, hospital based; M, male; PB, population based.

^*^Risk estimate was first recalculated by the method proposed by Danesh *et al*^.30^ and then summarized by the inverse-variance method.

**Table 2:** Summary risk estimates of the association between dietary fiber intake and pancreatic cancer risk.

	**No. of Studies**	**Summary risk estimate**	**95% CI**	***I*^2^ (%)**	***P*_*h*_†**	***P*_*h*_‡**
**Overall**	14	0.54	(0.44–0.67)	41.4	0.043	
						
**Study design**						0.093
Cohort study	1	1.01	(0.59–1.74)	N/A	N/A	
Case-control study	13	0.53	(0.46–0.62)	31.9	0.114	
						
Fiber type*						0.570
Soluble fiber	2	0.52	(0.37–0.73)	0	0.326	
Insoluble fiber	2	0.49	(0.36–0.66)	0	0.898	0.796§
Crude fiber	3	0.55	(0.30–1.02)	65.0	0.057	0.792§
						
Subgroup analyses*						
**Type of control subjects**						0.144
PB-CC	10	0.57	(0.45–0.71)	28.2	0.168	
HB-CC	3	0.41	(0.30–0.56)	0	0.410	
						
**Proxy respondent**						0.578
Yes	7	0.55	(0.40–0.76)	44.9	0.069	
No	6	0.52	(0.42–0.63)	14.6	0.321	
						
**Exposure assessment**						0.440
Validated FFQ/DHQ	8	0.51	(0.42–0.61)	0	0.517	
Others	5	0.58	(0.37–0.89)	56.4	0.032	
						
**Geographic location**						0.942
North America	6	0.57	(0.42–0.79)	49.4	0.065	
Europe	4	0.43	(0.30–0.62)	1.4	0.385	0.342¶
Asia	2	0.57	(0.40–0.80)	0	0.841	0.982¶
Oceania	1	0.26	(0.12–0.58)	N/A	N/A	0.208¶
						
Adjustment for confounders or risk factors*						
**BMI**						0.976
Yes	3	0.53	(0.40–0.70)	24.7	0.265	
No	10	0.52	(0.39–0.68)	38.2	0.086	
						
**Sex**						0.924
Yes	12	0.52	(0.42–0.64)	36.7	0.082	
No	1	0.54	(0.28–1.06)	N/A	N/A	
						
**Diabetes**						0.620
Yes	3	0.45	(0.26–0.76)	64.0	0.062	
No	10	0.53	(0.44–0.64)	26.6	0.183	
						
**Total energy consumption**						0.329
Yes	12	0.52	(0.44–0.61)	0	0.537	
No	1	0.65	(0.13–3.22)	N/A	N/A	
						
**Alcohol consumption**						0.533
Yes	5	0.47	(0.30–0.74)	62.0	0.022	
No	8	0.56	(0.46–0.68)	0	0.607	

BMI, body mass index; CI, confidence interval; FFQ, food frequency questionnaire; HB-CC, hospital-based case-control study; N/A, not available; PB-CC, population-based case-control study.

^*^Cohort study was excluded from the analysis.

^†^*P* value for heterogeneity within each subgroup.

^‡^*P*-value for heterogeneity between subgroups.

^§^The result of soluble fiber was treated as the reference group.

^¶^The result of North America was treated as the reference group.
