# Supplementary material for: Dietary fiber intake and pancreatic cancer risk: a meta-analysis of epidemiologic studies
Source: Sci Rep. 2015 Jun 2;5:10834. doi: 10.1038/srep10834 (PMC4451698; doi:10.1038/srep10834)
Supplement: Supplementary Information [file srep10834-s1.pdf]

Dietary fiber intake and pancreatic cancer risk: a meta-analysis of epidemiologic studies

Chun-Hui Wang, Chong Qiao, Ruo-Chen Wang, Wen-Ping Zhou

Supplementary Table S1. Methodological quality of cohort studies included in the meta-analysis*

First author (reference), publication year	Representativeness of the exposed cohort	Selection of the unexposed cohort	Ascertainment of exposure	Outcome of interest not present at start of study	Control for important factor or additional factor†	Assessment of outcome	Follow-up long enough for outcomes to occur‡	Adequacy of follow-up of cohorts§
Stolzenberg-Solomon et al [9], 2002	☆	☆	☆	☆	☆	☆	☆	☆

* A study could be awarded a maximum of one star for each item except for the item Control for important factor or additional factor. The definition/explanation of each column of the Newcastle-Ottawa Scale is available from (http://www.ohri.ca/programs/clinical_epidemiology/oxford.asp.).

† A maximum of 2 stars could be awarded for this item. Studies that controlled for cigarette smoking received one star, whereas studies that controlled for other important confounders such as body mass index (BMI), diabetes mellitus (DM) received an additional star.

‡ A cohort study with a follow-up time >10 y was assigned one star.

§ A cohort study with a follow-up rate >75% was assigned one star.

Supplementary Table S2. Methodological quality of case-control studies included in the meta-analysis*

First author (reference), publication year	Adequate definition of cases	Representativeness of cases	Selection of control subjects	Definition of control subjects	Control for important factor or additional factor†	Exposure assessment	Same method of ascertainment for all subjects	Non-response Rate‡
Bidoli et al [5], 2011	☆	☆	—	☆	☆☆	☆	☆	☆
Jansen et al [6], 2011	☆	☆	—	☆	☆☆	☆	☆	—
Zhang et al [7], 2009	☆	☆	☆	☆	☆	☆	☆	☆
Chan et al [8], 2007	☆	☆	☆	☆	☆☆	☆	☆	☆
Lin et al [3], 2005	☆	☆	☆	☆	☆	☆	☆	—
Ji et al [10], 1995	☆	☆	☆	☆	☆	—	☆	—
Lyon et al [12], 1993	☆	☆	☆	☆	☆	—	☆	—
Kalapothaki et al [11], 1993	☆	☆	—	☆	☆☆	—	☆	☆
Zatonski et al [16], 1991	☆	☆	☆	☆	☆	—	☆	—
Ghadrian et al [15], 1991	☆	☆	☆	☆	☆	—	☆	—
Mesquita et al [14], 1991	☆	☆	☆	☆	☆	☆	☆	—
Baghurst et al [13], 1991	☆	☆	☆	☆	☆☆	☆	☆	—
Howe et al [17], 1990	☆	☆	☆	☆	☆	—	☆	—

* A study could be awarded a maximum of one star for each item except for the item Control for important factor or additional factor. The definition/explanation of each column of the Newcastle-Ottawa Scale is available from (http://www.ohri.ca/programs/clinical_epidemiology/oxford.asp.).

† A maximum of 2 stars could be awarded for this item. Studies that controlled for cigarette smoking received one star, whereas studies that controlled for other important confounders such as body mass index (BMI), diabetes mellitus (DM) received an additional star.

‡ One star was assigned if there was no significant difference in the response rate between control subjects and cases by using the chi-square test (P>0.05).
